# Anaerobic digestion is the dominant pathway for pit latrine decomposition and is limited by intrinsic factors

**DOI:** 10.2166/wst.2019.220

**Published:** 2019-06-25

**Authors:** Miriam H. A. van Eekert, Walter T Gibson, Belen Torondel, Faraji Abilahi, Bernard Liseki, Els Schuman, Colin Sumpter, Jeroen H. J Ensink

**Affiliations:** 1 **Miriam H. A. van Eekert** (corresponding author) **Els Schuman** LeAF, Bornse Weilanden 9, 6708 WG Wageningen, The Netherlands; 2 **Walter T. Gibson** Bear Valley Ventures, Utkinton Lane, Tarporley, Cheshire CW6 0JH, UK; 3 **Belen Torondel Colin Sumpter Jeroen H. J. Ensink** Environmental Health Group, Faculty of Infectious and Tropical Diseases, London School of Hygiene and Tropical Medicine, Keppel Street, WC1E 7HT London, United Kingdom; 4 Faraji Abilahi Bernard Liseki Ifakara Health Institute, Off Mlabani Passage, P.O. Box 53, Ifakara, Tanzania

**Keywords:** biogas, COD removal, decomposition, pit latrine

## Abstract

*In vitro* methods were used to assess the full potential for decomposition (measured as biogas formation) from pit latrine samples taken from the top layer of 15 Tanzanian latrines. We found considerable variability in the decomposition rate and extent. This was compared with decomposition in the same latrines, measured by comparing top layer composition with fresh stools and deeper (older) layers, to assess whether this potential was realised *in situ*. Results showed a close match between the extent of organic material breakdown *in* situ and *in vitro,* indicating that anaerobic digestion is the dominant pathway in latrines. The average potential decrease in chemical oxygen demand (COD) (determined as methane production* in vitro* within 60 days) and actual measured decrease *in situ* are 68.9% ± 11.3 and 69.7% ± 19.4, respectively. However in the *in vitro* tests, where samples were diluted in water, full decomposition was achieved in 2 months, whereas *in situ* it can take years; this suggests that water addition may offer a simple route to improving latrine performance. The results also allowed us to estimate, for the ﬁrst time to our knowledge using experimental data, the contribution that latrines make to greenhouse gas emissions globally.This amounts to ∼2% of annual US emissions.

## INTRODUCTION

For most of the world’s poor, the only available option for sanitation is a pit latrine, and an estimated 1.77 billion people rely on them on a daily basis (Graham & Polizzotto 2013). Pit latrines are relatively easy and cheap to construct, but have several disadvantages, mostly linked to filling up with faecal waste and its slow stabilisation. Smells, flies, and cost or lack of emptying services all pose problems for users (Thye *et al*. 2011; Murungi & van Dijk 2014; Nakagiri *et al*. 2016). The time taken for a pit latrine to fill up is highly variable (Nakagiri *et al*. 2016) but qualitative research suggests it can be as little as 2 years (Zeeshan Ijaz *et al*. in preparation; Biran 2010).

Extending the lifetime of a pit latrine would thus be a major benefit to users.

The speed with which pit latrines fill up depends on a number of factors, which include the pit dimensions, number of users, the amount of excreta (urine and faeces) produced per person per day, drainage, and the rate of decomposition of excreta. Attempts to create a model combining these different factors in order to simulate actual fill-up rates have been made (Nwaweri *et al*. 2008) but have not been fully successful, which possibly reflects the lack of good available data on key parameters. The rate and extent of decomposition are key variables, and indeed for a given family in a particular location may be the only variables they can really control, for example through alteration of the latrine environment.

Previous studies on decomposition in pit latrines have been quite limited. Decreases in chemical oxygen demand (COD) levels with depth have been reported (Nwaweri *et al*. 2008). The bulk of the digestion would be expected to be anaerobic and it has been shown that latrine samples are capable of producing methane *in vitro* (Couderc *et al*. 2008). However, a model proposing some aerobic breakdown at the surface of latrines has been put forward (Buckley *et al*. 2008) and so there is still some uncertainty about the pathways responsible for stabilisation of organic matter in latrines and whether anaerobic digestion is mostly responsible.

Further experimental data on anaerobic digestion in latrines is also important because a number of authors have made estimates of methane production from latrines based on life cycle analysis or other theoretical approaches (Reid *et al*. 2014; Kulak *et al*. 2017) and these have suggested that it constitutes a significant contribution to global greenhouse gas emissions. These models have not yet been verified with experimental data on methane production.

Our objectives were therefore to evaluate the full potential of anaerobic processes in terms of the rate and extent of breakdown of latrine organic material, using laboratory studies, and to evaluate the extent to which this potential is achieved *in situ*. To our knowledge this is a novel approach. The data we obtained has significance both for the understanding of decomposition of latrines and how it can be accelerated, and also for the understanding of the contribution latrines make to global warming.

## MATERIAL AND METHODS

### Study area and latrine selection

The study was conducted in the Morogoro region of southern-central Tanzania, in the town of Ifakara and the villages of Signali and Sululu, roughly 10 km to the north of Ifakara. In rural areas of mainland Tanzania, 71% of households use unimproved toilet facilities, usually an unimproved pit latrine (Tanner *et al*. 1987). In January 2011 (outside of the rainy season) a total of 45 pit latrines were selected in the villages of Sululu and Signali, and the town of Ifakara. Latrines were selected to represent a wide diver-sity of design and use and included: lined, unlined, rural, urban, water users, non-water users, low intensity usage (<6 people/latrine), and high intensity usage (>15 people/ latrine).

### Stool survey

At the time of pit latrine sample collection, a stool survey was conducted among 15 selected households (which were included in the 45 pit latrines mentioned above). The households were selected based on geographical location (close to the laboratory) and willingness of the inhabitants to cooperate. Each household was provided with a blue and a red 10 L bucket with a lid, and all household members were asked for the duration of 1 day (24 hours) to use the buckets instead of the pit latrine for their daily defecation. Men were asked to use blue buckets, while women and children below the age of 12 were asked to use the red buckets. Buckets were numbered, and pre-weighed before distribution to households. A bin-liner was provided in each of the buckets to facilitate emptying and safe disposal following the experiment. Exactly 24 hours later buckets were collected, weighed, and following mixing, two approximately 150 mL samples were collected from each bucket. Mixing was performed manually with sterile stirrers. Each sample bottle was transported and stored on ice before analysis in the laboratory. Stool samples were analysed for the same parameters and using the same procedures as for pit latrine samples.

### Sample collection and initial sample analysis of stools and top layers of latrines

Latrine samples at 20 cm intervals were collected from the top of the pit to the bottom of the pit through the middle (below the drop hole) of the latrine. Depending on the con-sistency of the top material, samples were collected either with a standard soil auger (Eijkelkamp, Giesbeek, The Netherlands) or, if the top layer was liquid, with a sterile 150 mL plastic container attached to the soil auger. Deeper layers in liquid pit latrines were collected with a specially developed sampler that could be opened in the lower layers and used to collect a sample with a scraper without it being contaminated by layers above (Torondel *et al*. 2015). *In situ* temperature and pH measurements were taken with a hand-held meter (HI 991003, Hanna Instruments, USA) before samples were placed in two sterile sample containers (100 mL) and transported in a cool box for further analysis.

The first sample container was analysed the same day in the Ifakara Health Institute laboratories for pH (hand-held meter HI 991003, Hanna Instruments, USA), total chemical oxygen demand (COD_total_), and volatile fatty acids (VFA). Moisture content, total solids (TS) and volatile solids (VS) were analysed according to standard methods (APHA 2005) at the end of each collection week (samples for this analysis were kept at 4°C). Samples were homogenised using a homogeniser pack (Powergen 500, Fisher, UK) in which 1 g was diluted in 20 mL of _dd_H_2_O (double distilled water). The main purpose of homogenisation was to disperse the latrine sample in water and form a homogeneous preparation which could be used for chemical analysis. After homogenisation and dilution the mixture was passed through a 0.45 μm filter (except for COD_total_ analysis). Samples were analysed using Hach Lange test kits and methods (Hach Lange Loveland, USA), for COD_total_ and soluble COD (COD_soluble_) using the dichromate method, and for VFA an esterification method as described earlier by Torondel *et al*. (2015). The second sample container was transported (while the temperature of the sample was kept below 7°C) to another laboratory (Wageningen, The Netherlands) and used for the biodegradation tests within 1 month. Analysis of the COD_total_ of the samples directly after arrival in Wageningen, in comparison with the values obtained in Ifakara, confirmed that no major changes in organic matter had occurred during transport, except in one case, latrine Q, which was excluded from the *in vitro*/ *in situ* comparison (data not shown).

### Anaerobic biodegradation assays

The biodegradation assays were conducted with the top layer samples from selected pit latrines. The aim of the biodegradation tests was to assess the maximum biogas production possible from the samples without any addition of external nutrients (thus with samples as received) in order to assess whether the microorganisms present in the top layers of the pit latrines were able to convert the organic material present. Tests were carried out in duplicate in 1 L bottles with a total liquid volume of 200 mL. Depending on the amount of material available the amount of pit latrine samples used in the tests was 10–16 g. Anaerobically prepared demineralised water was added to each bottle to a final total (sample and water) weight of 200 g. Nutrients or buffer solutions were not applied in these tests. The tests were set up in an anaerobic hood to prevent contact of pit latrine samples with oxygen. At the start of the experiment the headspace of all bottles was changed to nitrogen gas (100%). All tests were incubated for a period of 14 days in a rotary shaker at 30°C (to avoid mass transfer limitation and speed up the conversion processes). Thereafter, the bottles were incubated statically in a temperature controlled compartment at 30°C. The incubation temperature was chosen based on the average temperature at the location during the complete sampling campaign, which was 25 ^o^C (19–36°C) (Torondel *et al*. 2015). At the time of sampling it was around 30–32°C. The total test period was 2 months. During the test, the gas production (and methane content) and pH were analysed on a regular basis. In most cases the pH remained between 6.5 and 8.0 and was not adjusted during the study.

Previous tests had shown that the methanogens in the pit latrine material are easily inactivated during sampling, transport and handling of the samples, leading to possible exposure to oxygen (data not shown). Accidental inactivation of this part of the microbial population could have inﬂuenced the outcome of the tests. Therefore, incubated bottles were checked regularly for biogas formation and in addition the methane content of the biogas was assessed twice (at day 14 and day 28) during the ﬁrst month. Tests were started up without the addition of an inoculum to assess intrinsic methanogenic activity. The necessity of inoculation with a methanogenic inoculum was determined after 14 days into the test, based on the biogas production and composition in the tests. Granular sludge (TS: 192 g/kg, VS: 73.3%) from an upﬂow anaerobic sludge blanket reactor treating paper mill wastewater was used as the methanogenic inoculum. The inoculum (5 grams wet weight) was added in one of the duplicate bottles if the biogas production and methane content were too low (pressure build-up less than 10 kPa and methane content below 50%). After 30 days of incubation the second (uninoculated) bottle of the duplicates was also inoculated if the biogas production and methane content were still low. If both bottles of a duplicate test had to be inoculated, the second bottle usually received a higher amount of methanogenic inoculum (10 grams wet weight) to ensure that anaerobic biodegradation was not hindered by the lack of the appropriate bacteria. A control test without substrate was included to correct for the endogenous biogas/methane production from the inoculum material.

TS, VS and total COD of top layer samples were deter-mined according to standard methods (APHA 2005). During the tests liquid samples were taken biweekly for pH, COD_soluble_ and VFA analysis. COD_soluble_ (determined in the supernatant after centrifugation of the samples at 10,000 rpm for 10 min) was analysed using Hach Lange test kits and a Hach Lange Xion 500 model LPG-385 spectrophotometer (Düsseldorf, Germany). Prior to VFA analysis the samples were centrifuged (10 min, 10,000 rpm) and diluted with formic acid to a ﬁnal concentration of 1.5%. VFA (C2 to C5 chain lengths) was determined in the supernatant using an HP 5890 gas chromatograph equipped with a glass column (2 m * 6 mm * 2 mm) packed with 10% Fluorad 431 on Supelco-port 100–120 mesh and a ﬂame ionisation detector. Nitrogen saturated with formic acid (40 mL/min) was used as a carrier gas. The temperature of the injector, oven and detector were 200, 130, and 280°C, respectively. The sample size was 1 μL. The detection limit for VFA analysis was 10 mg/L for each separate volatile fatty acid.

The biogas composition during and after the test was determined in a 50 μl sample using a Shimadzu GC 2010 equipped with loop injection and two columns operated in parallel. The columns used were a Porabond Q (50 m × 0.53 mm; 10 μm, Varian; Part. no. CP7355) for CO_2_ analysis and a Molsieve 5A (25 m × 0.53 mm; 50 μm, Varian; Part. no. CP7538) for O_2_, N_2_ and CH_4_. The injector and oven were operated at 120, and 75°C, respectively. Helium (pressure 1.0 bar) was used as a carrier gas. The thermal conductivity detector was kept at 150°C.

### Data analysis and calculations

The methane production (in g COD/ kg wet weight (ww)) in the laboratory tests was calculated from the biogas production (L/kg ww assessed at 30°C) using the following calculation:

MP ¼ BP _ %CH4=100 _16 _4=24:88

(with MP ¼ methane production in g COD=kg ww;

BP ¼ biogas production at 30_C in L=kg ww;

16 ¼ molar weight of CH4ðg=molÞ; 4 ¼ g COD=g CH4; and 24:88 ¼ molar gas volume at 30 _C in L=mol):

The potential COD removal was calculated as follows:

COD potential (%) ¼

100*((COD_stool_ - COD_top_) þ MP)=COD_stool_

The actual COD removal in situ was determined as follows:

COD actual (%) ¼ 100*(1 - COD_bottom_=COD_stool_):

For this calculation only the pit latrines where there was a depth of sludge of 0.8 to 1 m were included so that they would be broadly comparable and provide the best chance of seeing the full extent of COD removal in situ. The samples from the base layer in these pits would be around 6–7 years old based on the observed fill rates.

The potential global CO2 emission from pit latrines was calculated using the data from the selection of pit latrines of which the stool input had been characterised (total 15), excluding those that were not active in the *in vitro* laboratory tests (latrines F, M, N, and Q). The average VS content of the stool of the remaining 11 pit latrines was 179 g VS/kg ww (i.e. fresh stool), and the average methane production in the laboratory of those latrines was 196 L CH_4_/kg VS (at 30°C), so 177 NL CH_4_/kg VS. Furthermore a yearly stool production of 99 kg ww/person (p) was used based on mean production of 271 g ww/(p·day)) taken from Ensink *et al*. (2012).

### Ethics

Village leaders in Signali and Sululu and the neighbourhood leader in Ifakara approved the study following meetings in which the study was explained and questions were answered. The review board of the Ifakara Health Institute (IHI), the National Institute for Medical Research (NIMR) in Tanzania, and the London School of Hygiene and Tropical Medicine (LSHTM) granted ethical approval for this study (IHI 14-2-10, NIMR 1143, LSHTM 5659). Community meetings were held to introduce the study, and all study participants provided written informed consent.

## RESULTS AND DISCUSSION

### Biogas production from latrine samples in vitro

**Figure 1:**
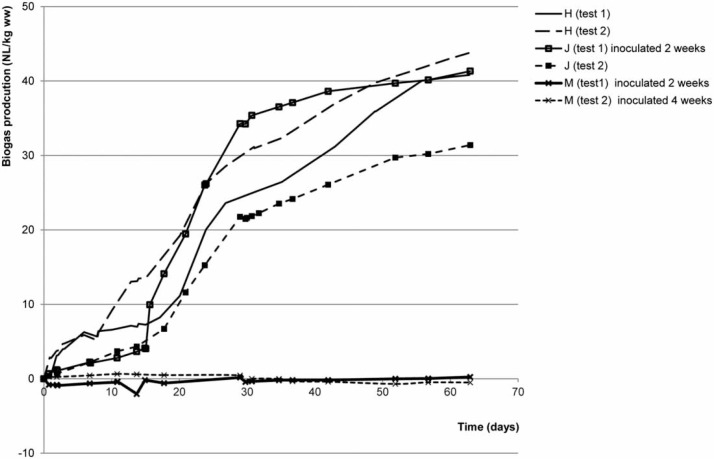
Biogas production in vitro in top layer samples taken from three different latrines. Shown are duplicate bottles of the same sample treated differently based on their performance as described in the ‘Material and methods’ section. ‘Inoculated 2 weeks’ and ‘inoculated 4 weeks’ indicate the time points at which granular sludge was added to promote methane formation. Other bottles did not receive an inoculum.

Previous studies have suggested that there is some rapid aerobic breakdown of easily biodegradable material at the surface of latrines (Nwaweri *et al*. 2008) and then it is expected that anaerobic processes will take over as the For this calculation only the pit latrines where there was a depth of sludge of 0.8 to 1 m were included so that they would be broadly comparable and provide the best chance of seeing the full extent of COD removal *in situ*. The samples from the base layer in these pits would be around 6–7 years old based on the observed ﬁll rates. layer of pit latrines were incubated under anaerobic conditions *in vitro* and biogas production followed. Three different patterns of biogas formation were observed. Examples of these patterns are shown in Figure 1, which shows cumulative biogas production against time.

Top layer latrine samples in Group 1 (e.g. sample H in Figure 1) started to form biogas immediately, with the major amount of the biogas being formed within 30–40 days. In Group 2 (e.g. sample J in Figure 1), a lag period was seen and an inoculum accelerated biogas formation but only shortened the lag period. In Group 3 (e.g. sample M in Figure 1), no biogas production was detected, even with an inoculum. This suggests that the available substrates in the top layers of these latrines were exhausted in the latter Group, which is borne out by the low values for VS/TS ratio in these samples (Table 1).

**Table 1:** Comparison between fresh stool and top layer in corresponding pit latrine (AV ¼ average; SD ¼ standard deviation)

		Fresh stool					Top layer sample (0–20 cm						
ID	Group	TS (g/kg)	VS (g/kg)	COD_total_ (g/kg)	VS/TS (-)	VFA (g/kg)	TS (g/kg)	VS (g/kg)	COD_total_ (g/kg)	VS/TS (-)	VFA (g/kg)	Delta VS/TS	Delta VS/TS as % of stool VS/TS
A	2	347	320	212	0.9	20	129	112	164	0.9	5	0	0
B	2	151	125	193	0.8	16	103	74	111	0.7	10	0.1	12.5
C	1	337	208	213	0.6	15	530	101	124	0.2	4	0.4	66.7
D	2	158	133	203	0.8	12	251	177	211	0.7	23	0.1	12.5
E	1	140	117	195	0.8	15	322	112	135	0.3	4	0.5	62.5
F	3	153	128	166	0.8	14	680	61	41	0.1	3	0.7	87.5
G	1	132	119	152	0.9	12	267	168	210	0.6	14	0.3	33.3
H	1	146	130	138	0.9	12	145	129	194	0.9	12	0	0
J	2	307	212	181	0.7	12	170	124	123	0.7	7	0	0
K	1	207	189	200	0.9	13	247	144	213	0.6	19	0.3	33.3
L	1	271	225	216	0.8	21	165	102	163	0.6	7	0.2	25
M	3	205	181	193	0.9	16	822	39	28	0.0	1	0.9	100
N	3	166	148	208	0.9	15	675	27	35	0.0	1	0.9	100
P	1	219	194	156	0.9	10	137	102	129	0.7	8	0.2	22.2
Q	3	60	46	74	0.8	10	564	49	79	0.1	4	0.7	87.5
AV		200	165	180	0.8	14	347	101	131	0.5	8		
SD		83	64	38	0.1	3	240	45	64	0.3	6		

Data from 45 different latrines (not shown) showed that the majority, around 73%, of latrines fell into Group 1, 9% in Group 2 and 18% in Group 3. These results show that the microbial communities and substrates required for anaerobic degradation are present in most pit latrines and that under the right conditions they can actively produce biogas. However the rates at which breakdown proceeds *in situ* are likely to be slower than *in vitro*. The top layer of 20 cm represents about 18 months of use, and in most cases there is still substantial potential for biogas production as revealed in the laboratory tests, whereas the laboratory tests (without inoculum) go to completion in a much shorter time, within 2 months. Only in the case of Group 3 does breakdown *in situ* appear to be rapid, with all the available substrates becoming depleted.

### Decomposition in *situ*

Breakdown was assessed by comparing the changes in key parameters, such as VFA content, COD content, and VS/TS ratio, from fresh stools to the top layer of latrines. In each case pooled stools from regular users were compared with samples taken from their latrine. Table 1 shows the results for 15 such comparisons. The VS/TS ratio is a broad measure of the amount of organic material remaining in the solid fraction of the latrine samples. By comparing this value in the stools to the corresponding value in the top layer of the latrine, the change in the amount of organic material can be followed. It is clear that there is considerable variability in this parameter between latrines, with the data suggesting that in some latrines (Group 3 types) there is virtually 100% removal of VS when the faeces arrive in the latrine, whereas in Group 1 VS removal was considerably lower (from 22–67%) and in Group 2 only 0–12.5% was removed. The underlying factors for the variability differ from group to group. In Group 1, there appears to be substrate and the required microbial communities are available but some kind of constraint is slowing the rate of breakdown *in situ* compared with the *in vitro* pattern. In the latter case conversion of the organic matter to biogas is mostly complete within 60 days, whereas in the former case the same extent of degradation requires a much longer time (from months to years; refer to next section for comparison). In the case of Group 2, whereas there is substrate available, the lag period observed *in vitro* suggests that some or all of the required microorganisms are not present in sufﬁcient numbers. In those pits where there is 0% breakdown taking place *in situ* but active breakdown after a lag *in vitro*, there is presumably some other environmental factor inhibiting decomposition. The nature of this factor was not investigated further in this study. A reason could be the presence of inhibiting compounds which are discharged in the latrine together with the latrine material (Nakagiri *et al*. 2016).

### Comparison of potential and actual breakdown

In order to assess whether the potential breakdown observed in laboratory tests is realised *in situ*, albeit more slowly, this was compared to the actual changes occurring from stool to bottom layer of the latrine. Only latrines where the bottom layers were at least 80 cm (4th layer) and of similar depth (4–5 layers, so 80–100 cm) were included in this analysis, which is shown in Table 2. This was to ensure that we considered latrines where the material was old enough to have undergone signiﬁcant stabilisation. Two further latrines, G and Q, were considered anomalous and not included in this analysis, G because the COD content of the top layer was greater than the fresh stool, suggesting that material other than faeces had been added, and Q because the COD content had reduced by about 60% on arrival in Wageningen. The potential changes in COD from stool to bottom layer, assuming that the full extent of *in vitro* breakdown was realised *in situ*, were taken as the sum of the measured change in COD from stool to top layer and the total amount of biogas formed from top layer samples *in vitro*, expressed as COD equivalents. The actual changes were taken as the difference in COD content between the stool and the bottom layer. As can be seen in Table 2, the average potential decrease in COD and the actual measured change are very similar, 68.9% ± 11.3 and 69.7% ± 19.4. This suggests that the biogas production *in vitro*, in terms of extent, does reﬂect the processes and changes occurring *in situ* and it is only the rate which differs. It is also the ﬁrst deﬁnitive experimental evidence for the widespread assumption that anaerobic digestion is the dominant pathway in pit latrines.

**Table 2:** Potential and actual removal of COD in pit latrines (SD ¼ standard deviation; SE ¼ standard error; ww ¼ wet weight)

		Top layer COD to CH4			Top layer COD to bottom COD	
ID	Stool to topCOD removedg/kg ww	CH4-COD producedg/kg ww	COD removal possible		COD bottom layer	
			g/kg ww	% of COD stool	g/kg ww	Actual removal in situ (%)
A	47.34	112.9	160.2	75.7	114.7	45.8
B	81.98	35.6	117.6	61.1	66.1	65.7
C	89.56	57.1	146.7	68.8	49.5	76.8
D	7.72	121.8	114.1	56.2	95.1	53.2
E	60.02	44.7	104.7	53.7	21.5	89.0
F	125.08	1.4	126.5	76.2	9.9	94.0
J	58.66	88.0	146.6	80.9	103.7	42.8
L	53.82	80.2	134.0	61.9	57.2	73.6
M	165.16	0.0	165.2	85.7	25.6	86.7
			Average	68.9	Average	69.7
			Median	68.8	Median	69.6
			SD	11.3	SD	19.4
			SE	3.8		6.5

Given that the main difference *in vitro* was the addition of excess water to the samples, this suggests that *in situ* the moisture content may be too low for optimal anaerobic digestion to proceed. This conﬁrms the earlier conclusions of Couderc *et al*. (2008), which were based on a much more limited latrine sample size (1) and solely *in vitro* testing. Other authors using high solids content sludge samples have shown that increasing the moisture content can increase methane production (Lay *et al*. 1997). It is possible that moisture content affects factors such as viscosity and nutrient and bacterial diffusion rates which will in turn affect metabolism.

Our results imply that latrine performance, in terms of solids reduction, could be enhanced substantially by controlling the moisture content. As an indication of the kind of change required, the average moisture content in the top layer of the pit latrines was 69% (range 18–100%) and in the laboratory the moisture content in the bottles was higher than 98.5% (10 to 16 grams of wet sample with average TS of 200 g/kg in total weight of 200 grams). Clearly water addition would need to be controlled to avoid simply filling the latrine with water. A further difference with respect to the *in vitro* tests was that the contents of the bottles were well mixed so there is no local accumulation of intermediates or end products. Latrine contents are not mixed at present and this could be a further factor to explore together with moisture content in future research.

In a larger study of 29 latrines where only top and bottom layer samples were compared, values of 47.8% ± 13.6 and 58.8% ± 28.2 COD removal were obtained for average potential removal and actual removal, respectively. These would be expected to be lower than the values from Table 1 as the change from stool to top layer was not included. Again there is a reasonably close comparison in the values given the variability present.

### Methane emissions from latrines

Given that the comparison of *in vitro* and *in situ* data is reasonably close and supports the view that anaerobic digestion is the dominant pathway of decomposition in pit latrines, we were then able to estimate the total methane emissions from pit latrines globally using the *in vitro* data. This is the ﬁrst time this has been done using direct experimental data of methane production, although others have modelled methane emissions from latrines and have suggested they make a signiﬁcant contribution to global greenhouse gas emissions (Reid *et al*. 2014; Kulak *et al*. 2017).

In making our estimate we assumed that the data presented here is representative of pit latrine emissions generally; given that anaerobic digestion is the main form of breakdown in latrines this is not an unreasonable assumption. Our calculation used the following additional assumptions:

(i) Most of the degradation takes place in the top 20 cm.
(ii) Urine drains away immediately (i.e. no contribution to the volume).
(iii) The average stool production per person per day is 271 g ww.
(iv) There are 1.77 billion latrines users in the world (Graham & Polizzotto 2013).
(v) Methane is not converted aerobically. All COD in stool is converted to methane.

Based on these assumptions it can be calculated that the yearly methane production potentially emitted in pit latrines per person is 3,140 NL/(p·year) (177 NL/kg VS * 0.179 kg VS/kg ww * 99 kg ww-stool/year). For all pit latrine users, that would amount to 5.6 * 10^9^ Nm^3^ CH_4_/year (worldwide), which equals 4.0 * 10^9^ kg/year (5.3 * 10^9^ * 16/22.414 (kmol/ kg)/(m^3^/kmol)), i.e. 4.0 million metric tonnes CH_4_ per year. This agrees quite well with the estimate from spatial modelling made by Reid *et al*. (2014) of 3.4 Tg methane per year in 2015.

With a global warming potential (GWP), the measure for the impact of a compound relative to CO_2_, of 28 (cumulative GWP over 100 years) (IPCC 2014) the annual CO_2_ emission of pit latrines amounts to 112 Mt CO_2_-equivalents. For comparison, the annual CO_2_ emission worldwide was 35,669 Mt in 2014 ( Joint Research Centre 2014), so pit latrines account for 0.3% of the emissions worldwide or, for example, 2% of the annual US emission. Although small relative to other sources, it is still signiﬁcant and may well increase: according to the latest estimates around 2.3 billion people do not have access to even basic sanitation (Unicef & WHO 2015). Meeting this challenge with pit latrines would more than double the burden of greenhouse gas emission from this source and accelerate global warming. In addressing this issue, urgent consideration should therefore be given to the application of technologies which mitigate greenhouse gas emissions, for example by burning biogas as a fuel or using aerobic or nutrient recovery treatment methods.

## CONCLUSIONS

The results show that the laboratory tests could be a useful tool to assess pit latrine performance. Biodegradation of organic matter in pit latrines was assessed *in vitro* and *in situ*. The extent of biodegradation observed in the laboratory matches the breakdown of COD observed in the ﬁeld. Therefore, the laboratory tests are a useful tool to assess the pit latrine performance with regards to stabilisation of the organic matter. As anaerobic conditions prevail in pit latrines, methane emissions from pit latrines worldwide may be very high; this may be a source of greenhouse gases that is presently overlooked but very signiﬁcant as they are estimated to be as high as 2% of the annual (2014) US CO_2_ emissions.
